# Identification of *Bartonella rochalimae* in Guinea Pigs (*Cavia porcellus*) and Fleas Collected from Rural Peruvian Households

**DOI:** 10.4269/ajtmh.19-0517

**Published:** 2019-10-28

**Authors:** María F. Rizzo, Lynn Osikowicz, Abraham G. Cáceres, Violeta D. Luna-Caipo, Segundo M. Suarez-Puyen, Ying Bai, Michael Kosoy

**Affiliations:** 1 Division of Vector-Borne Diseases, Centers for Disease Control and Prevention, Fort Collins, Colorado;; 2 Sección de Entomología, Instituto de Medicina Tropical “Daniel A. Carrión” y Departamento Académico de Microbiología Médica, Facultad de Medicina, Universidad Nacional Mayor de San Marcos, Lima, Peru;; 3 Laboratorio de Entomología, Instituto Nacional de Salud, Lima, Peru;; 4 Dirección Ejecutiva de Salud Ambiental, Sub Región de Salud de Cutervo, Dirección Regional de Salud Cajamarca, Cajamarca, Peru;; 5 Dirección Ejecutiva de Salud Ambiental y Control Vectorial, Red de Salud Utcubamba, Dirección Regional de Salud Amazonas, Amazonas, Peru

## Abstract

In the present study, we tested 391 fleas collected from guinea pigs (*Cavia porcellus*) (241 *Pulex* species, 110 *Ctenocephalides felis*, and 40 *Tiamastus cavicola*) and 194 fleas collected from human bedding and clothing (142 *Pulex* species, 43 *C. felis*, five *T. cavicola*, and four *Ctenocephalides canis*) for the presence of *Bartonella* DNA. We also tested 83 blood spots collected on Flinders Technology Associates (FTA) cards from guinea pigs inhabiting 338 Peruvian households. *Bartonella* DNA was detected in 81 (20.7%) of 391 guinea pig fleas, in five (2.6%) of 194 human fleas, and in 16 (19.3%) of 83 guinea pig blood spots. Among identified *Bartonella* species, *B. rochalimae* was the most prevalent in fleas (89.5%) and the only species found in the blood spots from guinea pigs. Other *Bartonella* species detected in fleas included *B. henselae* (3.5%), *B. clarridgeiae* (2.3%), and an undescribed *Bartonella* species (4.7%). Our results demonstrated a high prevalence of zoonotic *B. rochalimae* in households in rural areas where the research was conducted and suggested a potential role of guinea pigs as a reservoir of this bacterium.

## INTRODUCTION

The Andes region of Peru, Colombia, and Ecuador is endemic for Carrión’s disease, a bacterial infection caused by *Bartonella bacilliformis*. This bacterium is transmitted to humans via the bite of a sand fly (*Lutzomyia verrucarum*). In addition to *B. bacilliformis*, other zoonotic *Bartonella* species (*B. rochalimae*, *B. ancashensis*, *B. henselae*, and *B. clarridgeiae*) have also been reported in Peru.^1–4^ Some of these *Bartonella* species are hosted by small carnivorous mammals (cats, dogs, skunks, and raccoons) and can be transmitted to humans by exposure to an infected animal and/or its ectoparasites.^5^ In humans, the symptoms associated with these *Bartonella* species are frequently nonspecific.^6–9^ For example, *B. rochalimae* isolated from an American tourist after her travel to Peru caused fever, bacteremia, and splenomegaly.^1^

People in small rural communities in the Andean and Amazonian region of Peru still live a very traditional lifestyle. As a result, they could be exposed to multiple zoonotic *Bartonella* species because of the frequent contact with various livestock and domestic animals. One type of livestock unique to these areas is guinea pigs (*Cavia porcellus*). They are kept for domestic consumption and sale, and are commonly used in folk medicine and religious ceremonies.^8–10^ Most guinea pigs are raised in the kitchen and can roam around freely in the household, whereas others are kept in pens adjacent to the houses. In addition to guinea pigs, other animals such as dogs, cats, and chickens can move freely inside and around the houses (G. A. Cáceres, personal communication). Traditionally, families in rural areas have an average of 20 guinea pigs per household.^11,12^ According to the Ministry of Agriculture and Irrigation of Peru, Peruvians alone consume an estimate of 65 million guinea pigs each year.^13^

In rural areas endemic for Carrión’s disease, diagnosis is typically based on clinical symptoms and Giemsa-stained blood smears. Although this assay has a high specificity, without proper training, the sensitivity can be as low as 36%.^14^ Diagnosis can also be determined based on blood culture of *B. bacilliformis*, which is best grown in 5% rabbit or sheep blood agar incubated at 28°C for up to 8 weeks.^14–16^ Other techniques, such as Western blot and polymerase chain reaction (PCR), are both more sensitive and specific, but the cost and lack of infrastructure restrict their use to larger laboratories in Lima.^17^ Techniques for diagnosing other *Bartonella* species rely on similar diagnostic tests but not on symptomatology.^15^

The objective of the present study was to identify *Bartonella* species circulating in guinea pigs and fleas within rural households from several parts of Peru. We used molecular techniques to test guinea pig blood spots and fleas found in households for *Bartonella* species.

## MATERIALS AND METHODS

### Ethics statement.

This research was conducted in compliance with the Animal Welfare Act and other federal statutes and regulations relating to animals and experiments involving animals and adheres to principles stated in the Guide for the Care and Use of Laboratory Animals, National Research Council Publication (1996 edition).

### Study sites.

**Figure 1.**
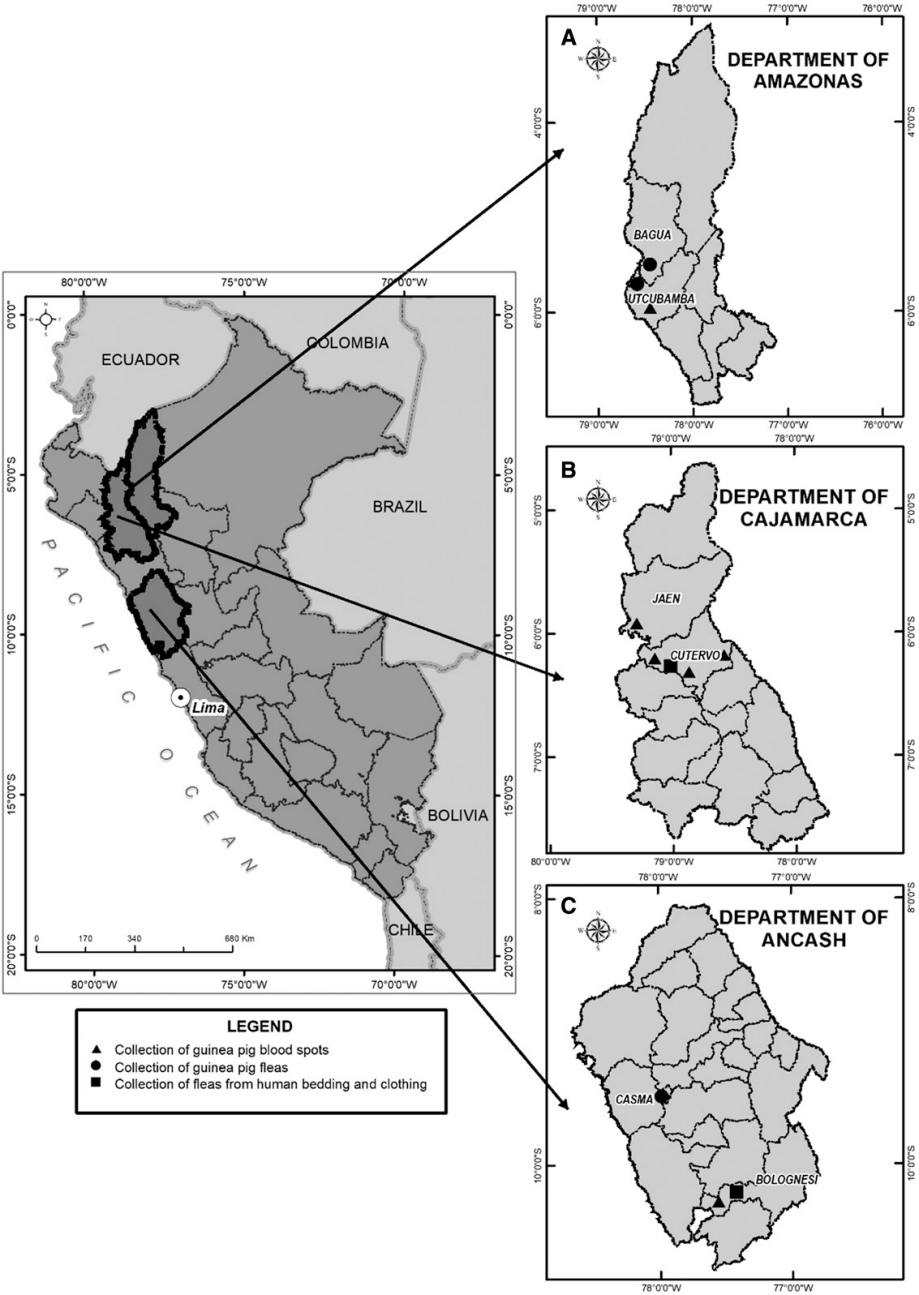
(**A**) Department of Amazonas: guinea pig blood was spotted on Whatman FTA cards in the province of Utcubamba, whereas guinea pig fleas were collected in Utcubamba and Bagua. (**B**) Department of Cajamarca: guinea pig blood was spotted on Whatman FTA cards in two provinces—Cutervo and Jaén, whereas fleas from human bedding and clothes were collected only in Cutervo. (**C**) Department of Ancash: guinea pig blood was spotted on Whatman FTA cards in Casma, and fleas from guinea pigs and human bedding and clothes were collected in Bolognesi.

The study was carried out in 36 localities of six provinces, belonging to the departments of Amazonas, Ancash, and Cajamarca (Figure 1) in 2012. These localities are between 466 and 2605 meters above sea level, and the average annual temperature varies from 12.6°C to 22.2°C. According to Brack–Egg’s classification of ecoregions, the localities belong to equatorial dry forest, highland steppes, and high jungle (cloud forest).^18^

In total, 338 households were selected by convenience for this study (4–9 houses per locality). The houses were built with adobe material with internal and external walls not pasted. A smaller number of houses had reed walls pasted with mud. All the houses had tin roofs and dirt floors throughout the rooms. The number of rooms per household varied from 2 to 4, including a kitchen/dining room, bedroom, and storage room. All of the houses were located in rural areas, close to farmland.

People raise guinea pigs in the kitchen of the houses. Other animals, including chickens, dogs, and cats, move freely inside the houses all day round. Outside the homes, people raise larger livestock, including turkeys, ducks, sheep, goats, rabbits, pigs, cattle, horses, and donkeys.

### Flea collection from guinea pigs.

Fleas were collected by hand from the guinea pigs and placed in 2-mL vials containing 70% alcohol. Each vial contained approximately 20 fleas. The vials were stored at room temperature at the Tropical Medicine Institute “Daniel A. Carrion” of the Universidad Nacional Mayor de San Marcos before shipment to CDC, Fort Collins, where they were identified and processed.

### Flea collection from human bedding and clothes.

Fleas were collected by hand from underwear, shirts, pants, socks, blankets, bed sheets, and pillowcases. For the purpose of our study, we combined the fleas for testing and defined them as fleas from human bedding and clothes.

### Guinea pig blood sampling on FTA cards.

Available blood of guinea pigs was spotted on Whatman Non-Indicating FTA Classic Cards (Whatman, GE Healthcare, Pittsburgh, PA). The FTA cards were then dried and kept at room temperature until shipped to CDC, Fort Collins, for processing. No inclusion/exclusion criteria were used for selecting the guinea pigs.

### Flea identification.

Fleas were identified to genus and species level using a dissecting microscope and taxonomic keys.^19,20^ Because of the continuing debate of taxonomic differences between *Pulex irritans* and *Pulex simulans*,^21^ fleas of these species were grouped by genus only and labeled as *Pulex* spp.

### Flea DNA extraction and PCR.

Individual fleas were placed in a 1.5-mL sterile Navy Snap Cap microcentrifuge tube (Next Advance, Troy, NY) with 400 mL of brain heart infusion media (BHI). One tube containing no flea but only BHI was included as a negative control. The tubes were placed in a Bullet Blender Gold homogenizer (Next Advance) for 15 minutes, centrifuged at 3,000 rpm for 1 minute, and 200 µL of the supernatant transferred to a new 1.7-mL sterile tube. DNA was extracted using a QIAxtractor (Qiagen, Valencia, CA) according to the tissue protocol provided by the manufacturer. A PCR assay was used to detect the presence of *Bartonella* spp. DNA by amplification of the 16S–23S intergenic spacer region (ITS) using primers 325f and 1100r as described by Diniz et al.,^22^ and the citrate synthase gene (*gltA*), using primers CS443f and CS1210r, as described by Birtles and Raoult.^23^ Nuclease-free water was used as a negative control and *Bartonella doshiae* DNA as a positive control. All PCR-positive samples were purified using a QIAquick PCR purification kit (Qiagen) according to the manufacturer’s instructions and sequenced with the Applied Biosystems 3130 Genetic Analyzer (Applied Biosystems, Foster City, CA). Sequences were analyzed using Lasergene 14 software (DNASTAR, Madison, WI), and phylogenetic analysis was performed using the CLUSTAL W alignment with Lasergene 14 software (DNASTAR). Only samples that were sequence positive with ITS and *gltA* were considered positive.

### Whatman FTA card DNA extraction and PCR.

DNA extractions were performed using QIAamp^®^ DNA Mini Kit extraction kits (Qiagen, Chasworth, CA) following the dried blood spot protocol provided by the manufacturer. A blank card was included in the extraction as a negative control. DNA samples were tested by PCR for the presence of *Bartonella* spp. using ITS and gltA and sequenced as described earlier. Only samples that were sequence positive with both ITS and *gltA* and the sequences of two targets that correspond to each other were considered positive.

## RESULTS

### Fleas.

A total of 585 fleas, including 391 (66.8%) collected from guinea pigs and 194 (33.2%) collected from human bedding and clothes, belonged to *Pulex* spp., *Tiamastus cavicola*, and *Ctenocephalides felis* and were tested by PCR for *Bartonella* DNA (Table 1).

**Table 1:** Flea distribution by province and host/source, 2012, Peru

Province	Flea species	From guinea pigs		From human bedding and clothes		Total	
		No. of fleas	%	No. of fleas	%	No. fleas	%
Bolognesi	*Pulex* sp.	10	2.6	66	34	76	13
	*Pulex* sp.	84	21.5	76	39.2	160	27.4
Cutervo	*Ctenocephalides felis*	104	26.6	43	22.2	147	25.1
	*Ctenocephalides canis*	0	0	4	2.1	4	0.7
	*Tiamastus cavicola*	33	8.4	5	2.6	38	6.5
Jaén	*Pulex* sp.	9	2.3	0	0	9	1.5
	*Tiamastus cavicola*	7	1.8	0	0	7	1.2
Utcubamba	*Pulex* sp.	138	35.3	0	0	138	23.6
	*Ctenocephalides felis*	6	1.5	0	0	6	1
Total		391	1	194	1	585	1

### *Bartonella* DNA in fleas from guinea pigs.

**Figure 2.**
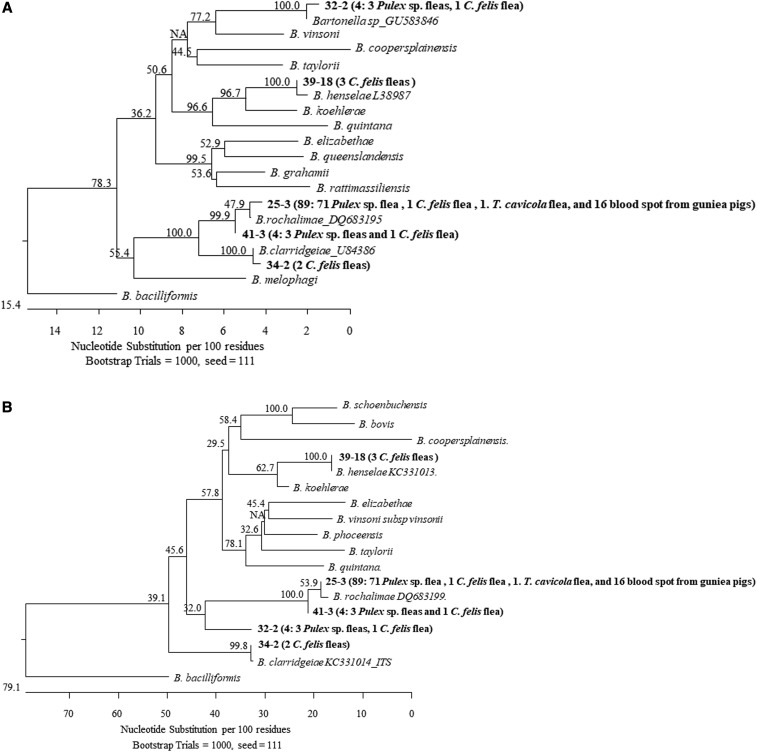
Phylogenetic relations of 102 sequences of partial *gltA* (**A**) and intergenic spacer region (**B**) of *Bartonella* DNA detected in fleas collected from guinea pigs or human bedding and clothes, and blood spots from guinea pigs. The phylogenetic tree was constructed by the N-J method and bootstrap values with 1,000 replicates. The majority (87.3%) of *Bartonella* sequences belonged to *Bartonella rochalimae*, within which two similar variants were identified; the other *Bartonella* sequences belonged to *Bartonella henselae*, *B. clarridgeiae*, and an unidentified *Bartonella* species. Sample ID in bold represents a particular variant identified in the present study. Following the ID is the number of identical sequences of that particular variant and the sources of the sequences.

*Bartonella* DNA were amplified from 81 fleas collected from guinea pigs, including 74 *Pulex* spp., one *T. cavicola*, and six *C. felis* (Table 2). Sequencing analyses showed that the *Bartonella* DNA in majority of the positive *Pulex* spp. belonged to *B. rochalimae* (71/74; 95.9%). The ITS sequences were either 100% identical or 99.7% similar to a previous described variant (DQ683199), whereas the *gltA* sequences also showed 99.7–100% similarity/identity to a previously described variant (DQ683195). The remaining three (4.1%) *Bartonella* DNA in *Pulex* spp. were identical to each other by both ITS and *gltA* but distant from other *Bartonella* spp., and presumably represent a new *Bartonella* species (Figure 2A and B).

**Table 2:** Distribution of *Bartonella*-infected fleas and guinea pigs (*Cavia porcellus*) and identification of the *Bartonella* species

Host	Total		From guinea pigs		From human bedding and clothes		*Bartonella* sp.
	Tested	Positive	Tested	Positive	Tested	Positive	
*Pulex* sp.	383	77	241	71	142	3	*B. rochalimae*
				3	–	–	*Bartonella* sp*.*
*Tiamastus cavicola*	45	1	40	1	5	0	*B. rochalimae*
*Ctenocephalides felis*	–	–	110	1	–	–	*B. rochalimae*
	153	8		3	–	–	*B. henselae*
	–	–		2	–	–	*B. clarridgeiae*
	–	–	–	–	43	1	*Bartonella* sp*.*
*Cavia porcellus*	83	16	–	–	–	–	*B. rochalimae*

Only one (2.5%) *T. cavicola* flea was positive, and the DNA sequences were identical to those found in *Pulex* fleas and belong to *B. rochalimae*.

Of the six *C. felis*–positive fleas, only one (16.7%) was positive for *B. rochalimae*. Three sequences (50%) belonged to *B. henselae* and were identical to KC331013 by ITS and L38987 by *gltA*. The last two (33.3%) sequences belonged to *B. clarridgeiae* and were identical to C331014 by ITS and U84386 by *gltA*.

### Bartonella DNA in fleas from human bedding and clothes.

A total of 190 samples were tested, and five (2.6%) fleas (three *Pulex* spp. and two *C. felis*) were positive for *Bartonella* DNA by ITS and *gltA* (Table 2).

The three positive *Pulex* spp. fleas belonged to *B. rochalimae* (identical to DQ683199 and DQ683195 by ITS and *gltA*, respectively). Of the two positive *C. felis* fleas, one belonged to *B. rochalimae* and the other one was identical to the new sequence identified in *C. felis* collected from guinea pigs mentioned earlier (Figure 2A and B).

### Bartonella DNA in blood spots from guinea pigs.

A total of 83 guinea pig blood spots on Whatman FTA cards were tested, and 16 (19.3%) samples were *Bartonella* DNA positive by both ITS and *gltA*. Sequencing analyses showed they belonged to *B. rochalimae* and were of the same genotype by each gene (identical to DQ683199 by ITS and DQ683195 by *gltA*) (Figure 2A and B).

## DISCUSSION

In the present study, *B. rochalimae* was the main *Bartonella* species found in fleas from guinea pigs and fleas from human bedding and clothes (89.5%) and the only *Bartonella* spp. found in guinea pig blood samples. *B. rochalimae* has previously been detected in several animals such as coyotes (*Canis latrans*), striped skunks (*Mephitis mephitis*), red foxes (*Vulpes vulpes*), and raccoons (*Procyon lotor*) from the United States,^5^ and brown rats (*Rattus norvegicus*) from Taiwan.^24^ Our results suggest that guinea pigs may be an additional animal reservoir for *B. rochalimae*.

The first description of *B. rochalimae* was in a *Pulex* spp. flea from Peru in 2002.^25^ Later *B. rochalimae* was isolated from an American tourist after her return home from a trip to Peru. The patient presented symptoms similar to those of Oroya fever (fever, bacteremia, and splenomegaly).^1^ In our study, *Pulex* spp. was the most abundant flea species, and 96% of the fleas tested were positive for *B. rochalimae*. This flea species often feeds on a variety of mammals, including guinea pigs, cats, dogs, and humans,^26–29^ and it is a known vector of plague and murine typhus.^30–32^ In these regions of Peru, where people live in close contact with guinea pigs, flea infestations of animals living in the household can be a source of exposure to zoonotic pathogens.

Some researchers have proposed that other *Bartonella* spp. besides *B. bacilliformis*, such as *B. rochalimae* and *B. ancashensis*, may be the cause of milder cases of Carrión’s disease.^17,33^ In 2014, Mujica et al.^34^ conducted a retrospective study where they identified *B. rochalimae* in the blood of a Peruvian patient originally diagnosed with Carrión’s disease. It can be difficult to diagnose *Bartonella* if diagnostic techniques rely on culture and clinical symptoms alone. *Bartonella* is known to be difficult to culture. They are slow growing and there are different growth requirements for *B. bacilliformis* (5% rabbit or sheep blood agar at 28°C) compared with other *Bartonella* spp., including *B. henselae*, *Bartonella elizabethae*, and *Bartonella quintana* (5% rabbit or sheep blood at 5% CO_2_ and 35°C). Moreover, some *Bartonella* spp., such as *B. rochalimae* and *B. clarridgeiae*, have an especially hard time growing on blood agar.^35^ These issues may result in a misdiagnosis or a missed diagnosis of *Bartonella*, and molecular testing should be considered as an additional diagnostic tool in these areas.

The remaining *Bartonella* spp. (*B. henselae* and *B. clarridgeiae*) found in the fleas from our study were mainly detected in *C. felis*, which is also known as the cat flea. Despite its common name, this flea readily feeds on other mammals available, including humans. Most likely, the positive *C. felis* from our study had previously fed on cats because it is known that cats are the host of *B. henselae* and *B. clarridgeiae*.^36–38^

The detection of *B. rochalimae* in guinea pigs, their fleas, and fleas from human bedding and clothes, as well as *B. henselae* and *B. clarridgeiae* from cat fleas should be taken into consideration when diagnosing *Bartonella* in these areas. Further research is needed to define the role of the guinea pigs and their ectoparasites in the transmission cycle in Peruvian rural households.
